# Comparison of non-laser and laser transvenous lead extraction: a systematic review and meta-analysis

**DOI:** 10.1093/europace/euad316

**Published:** 2023-10-26

**Authors:** Zaki Akhtar, Christos Kontogiannis, Georgios Georgiopoulos, Christoph T Starck, Lisa W M Leung, Sun Y Lee, Byron K Lee, Sreenivasa R K Seshasai, Manav Sohal, Mark M Gallagher

**Affiliations:** Department of Cardiology, St George’s University Hospital, London, UK; Department of Cardiology, St George’s University Hospital, London, UK; School of Biomedical Engineering and Imaging Sciences, King’s College London, London, UK; Department of Clinical Therapeutics, National and Kapodistrian University of Athens, Athens, Greece; Department of Cardiothoracic and Vascular Surgery, German Heart Center Berlin, Berlin, Germany; Department of Cardiology, St George’s University Hospital, London, UK; Department of Medicine, San Joaquin General Hospital, French Camp, CA, USA; Division of Cardiology, University of California, San Francisco, CA, USA; Department of Cardiology, St George’s University Hospital, London, UK; Department of Cardiology, St George’s University Hospital, London, UK; Department of Cardiology, St George’s University Hospital, London, UK

**Keywords:** Transvenous lead extraction, Lead extraction, Laser lead extraction, Non-laser lead extraction, Rotational lead extraction

## Abstract

**Aims:**

Transvenous lead extraction (TLE) is performed using non-laser and laser techniques with overall high efficacy and safety. Variation in outcomes between the two approaches does exist with limited comparative evidence in the literature. We sought to compare non-laser and laser TLE in a meta-analysis.

**Methods and results:**

We searched Medline, Embase, Scopus, ClinicalTrials.gov, and CENTRAL databases for TLE studies published between 1991 and 2021. From the included 68 studies, safety and efficacy data were carefully evaluated and extracted. Aggregated cases of outcomes were used to calculate odds ratio (OR), and pooled rates were synthesized from eligible studies to compare non-laser and laser techniques. Subgroup comparison of rotational tool and laser extraction was also performed. Non-laser in comparison with laser had lower procedural mortality (pooled rate 0% vs. 0.1%, *P* < 0.01), major complications (pooled rate 0.7% vs. 1.7%, *P* < 0.01), and superior vena cava (SVC) injury (pooled rate 0% vs. 0.5%, *P* < 0.001), with higher complete success (pooled rate 96.5% vs. 93.8%, *P* < 0.01). Non-laser comparatively to laser was more likely to achieve clinical [OR 2.16 (1.77–2.63), *P* < 0.01] and complete [OR 1.87 (1.69–2.08), *P* < 0.01] success, with a lower procedural mortality risk [OR 1.6 (1.02–2.5), *P* < 0.05]. In the subgroup analysis, rotational tool compared with laser achieved greater complete success (pooled rate 97.4% vs. 95%, *P* < 0.01) with lower SVC injury (pooled rate 0% vs. 0.7%, *P* < 0.01).

**Conclusion:**

Non-laser TLE is associated with a better safety and efficacy profile when compared with laser methods. There is a greater risk of SVC injury associated with laser sheath extraction.

## Graphical Abstract

**Graphical Abstract:**
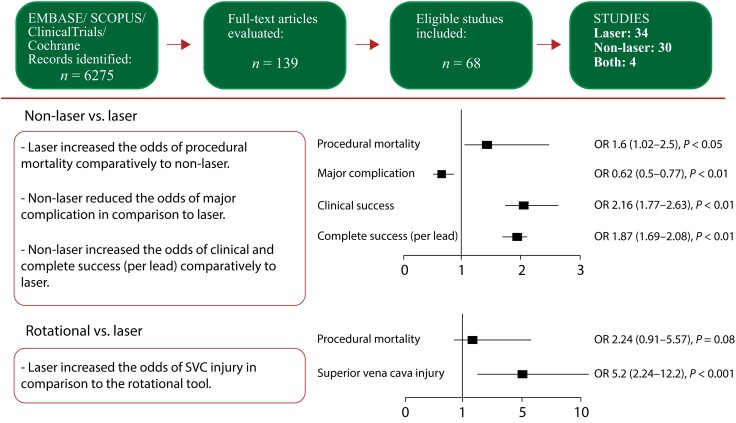

**What’s new?**

- This is the largest meta-analysis to date comparing non-laser and laser transvenous lead extraction.
- Non-laser transvenous lead extraction in comparison with laser is associated with better clinical and complete success with a lower complication risk, including superior vena cava injury and procedural mortality.
- Laser sheath extraction potentially carries a five-fold significantly greater risk of superior vena cava injury than rotational sheath extraction.
- Rotational sheath-assisted lead extraction is associated with higher clinical and complete success rate than laser.

## Introduction

The implantation rate for cardiac implantable electronic devices (CIEDs) continues to increase.^1,2^ They are a cornerstone of cardiac rhythm management and improve outcomes in heart failure. With this long-term reliance on CIEDs, transvenous lead extraction (TLE) has become integral to the management of cardiovascular disease, usually necessitated by device infection or hardware malfunction.^1^

The removal of leads is impeded by the scar tissue that forms at points of contact with the vessel wall, eventually encapsulating the leads. The tissue solidifies and calcifies binding leads to each other and to the wall of vessels and to the myocardium. Binding to the superior vena cava (SVC) is particularly relevant. To free leads from this binding, laser energy can be used to disintegrate the adhesions. Non-laser extraction alternatives include the use of dilating polypropylene sheaths to carefully dissect scar tissue around the lead as traction is applied. More recently, rotational sheaths have largely superseded other methods in non-laser TLE.

Both laser and non-laser techniques are efficacious and relatively safe,^3–6^ but there is limited data directly comparing the techniques. We aim to compare these two techniques in terms of safety and efficacy through a systematic review and meta-analysis of prospective and retrospective clinical studies.

## Methods

### Search strategy

This meta-analysis was registered to PROSPERO (ID: CRD42022291773) and was conducted in accordance with the Meta-analysis of Observational Studies in Epidemiology (MOOSE) proposal and Preferred Reporting Items for Systematic Reviews and Meta-Analysis (PRISMA) statement.^7,8^ We searched the Medline (PubMed), Embase, Scopus, ClinicalTrials.gov, and Cochrane Library—Cochrane Central Register of Controlled Trials (CENTRAL) databases for clinical studies published between January 1991 and March 2021 that reported the use of laser and non-laser techniques for TLE. A search was performed for relevant studies using Boolean operators in combination with the terms: ‘lead extraction’, ‘transvenous lead extraction’, ‘pacemaker lead extraction’, ‘defibrillator lead extraction’, ‘cardiac implantable electronic device extraction’, ‘mechanical lead extraction’, ‘laser lead extraction’, ‘non laser lead extraction’, ‘laser’, and ‘rotational’.

The titles and abstracts of the search results were screened independently by two authors to identify eligible studies based on a pre-defined inclusion criterion. The selected studies were compared, and any disagreements were adjudicated by a senior author, ensuring consensus was reached with a resolution being reached with consensus. Having identified the relevant studies, full articles were obtained and assessed for data extraction. We then manually searched the references of the retrieved articles, as well as of the related letters and editorials, to identify potential missed studies. All abstracts from large international congresses were also sought and screened. Quality assessment of the retrieved articles was performed using a methodological tool utilized by previous meta-analyses as well as the National Institute of Clinical Excellence (see Supplementary material online, *Table S1*).^9^

### Study criteria

Published prospective and retrospective studies reporting the use of non-laser or/and laser-powered sheaths for TLE were included in this meta-analysis without language restrictions. Studies were excluded if they met any of the following criteria: 1) studies irrelevant to the subject or outside of the study period, 2) reviews and meta-analyses, 3) case reports and case series or studies with a sample size of fewer than 20 patients, 4) letters to the editor, perspectives and editorials, 5) studies in which methods of TLE were not clearly specified, 6) studies with incomplete data, 7) and studies of exclusively paediatric (<18 years of age) cohorts.

### Data extraction

Relevant articles were assessed independently by two authors to extract data regarding year of publication, study period, institution where the extractions were performed, population size, baseline characteristic including age and gender, number of leads targeted for extraction, lead dwell time, proportion of infection indication, the method of extraction, type of sheath used for the extraction, major and the minor complications, procedural and non-procedural mortality, the 30-day mortality, complete procedural success rate, and the clinical success rate. All available mortality data were also adjudicated by the same two authors; disagreements were settled by the lead author.

Procedural success and complications were defined in accordance with the European Heart Rhythm Association (EHRA) and Heart Rhythm Society (HRS) consensus. Complete procedural success was defined as the successful removal of all components of the targeted lead without resulting in permanent disabling complications or death. Clinical success was defined as the achievement of the intended procedure endpoints with the removal of all parts of all leads other than a small lead component (<4 cm), in the absence of disabling complications or mortalities. An undesired consequence of the procedure that required medical or a minor procedural intervention was classed as a minor complication; a major complication was classified as an undesired consequence of the procedure that was life-threatening, resulted in death or caused significant disability.^1,10^ Procedural mortality was accepted as the occurrence of death arising from a major complication of the TLE, during or after the procedure.

To avoid duplication, studies from the same institution were evaluated, and if the research periods overlapped, only the larger study was included. In cases where a study compared two or more groups, each group was treated as an independent study; this approach was also applied to studies that reported on both laser and non-laser techniques. To maintain consistency, all studies were analysed for their stated complications as there was a degree of heterogeneity in the definitions. Studies that did not conform to the definitions of minor and major complications as outlined by the EHRA and HRS statements^1,10^ were analysed carefully by the authors to recalculate the numbers, with all decisions verified by at least two authors. Narrated major complications were evaluated carefully to verify the attributed causative technique, and subsequently, the major complication numbers for the relevant technique were updated accordingly. This methodology was applied to all included studies for consistency.

As the occurrence of a permanently disabling complication or procedural mortality constitutes a procedural failure based on present definitions, the success rates of older studies were adjusted to adhere to the current definitions of clinical and complete success. To obtain an accurate number for procedure-related mortality, reported deaths were analysed from the included studies and were accepted as ‘procedural related’ if there was unequivocal association between the procedure and death, and the death had occurred in hospital.

### Statistical analysis

With respect to safety and efficacy outcomes, we aggregated all cases (i.e. deaths, complications, and success) and non-cases from eligible studies per technique and calculated the odds ratio (OR) of death for laser vs. non-laser extraction. Next, we synthesized eligible studies and compared the pooled estimate between the two techniques separately for the following:

- rate of complete success (per lead) and clinical success (per patient);
- rate of in-hospital mortality and procedural mortality; and
- rate of major and minor complications.

The inverse hyperbolic tangent was used for the *r*-to-*Z* transformation of rates of interest before the pooled analysis of each corresponding outcome. We employed the robust Paul–Mandel heterogeneity estimator in random-effects models. The two techniques (laser vs. non-laser) were compared in terms of rates of interest by the test for heterogeneity between subgroups (*P* for interaction). The mean effect size and confidence interval (CI) of individual studies were illustrated with forest plots. We used the *I*^2^ measure to quantify heterogeneity.

**Figure 1:**
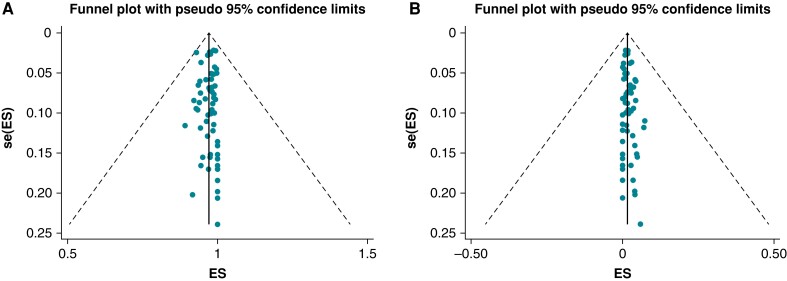
Funnel plots for publication bias. For the two main outcomes of efficacy and safety [successful extraction per patient (*A*) and occurrence of major complications (*B*)], we did not find evidence of major asymmetry in funnel plots or small-study effect (*P* > 0.05 for both Egger and Begg’s test in both endpoints). Circles represent individual studies of the meta-analysis and the vertical line the pooled estimate of the rate of (*A*) complete success per patient (*B*) major complications.

To further explore sources of heterogeneity, we applied random-effects meta-regression and assessed the effect of available continuous moderators (prevalence of traditional risk factors, i.e. hypertension and diabetes, mean age of participants, mean ratio of male to female subjects, and the year of study performance) on the difference of the rate of success between the two techniques. We conducted a pre-specified sensitivity analysis for the main outcomes of interest (rate of success and complications) by excluding older studies (i.e. before 2010). We explored the presence of publication bias graphically by funnel plots (*Figure 1*) of precision and statistically by regression tests for asymmetry (i.e. the Egger test and the Begg and Mazumdar test).

Statistical analysis was performed with the STATA package, version 12.1 (StataCorp, College Station, TX, USA). The module ‘admetan’ was used for meta-analysis in STATA. All tests were two tailed. We set statistical significance at *P* < 0.05.

## Results

### Literature search

**Figure 2:**
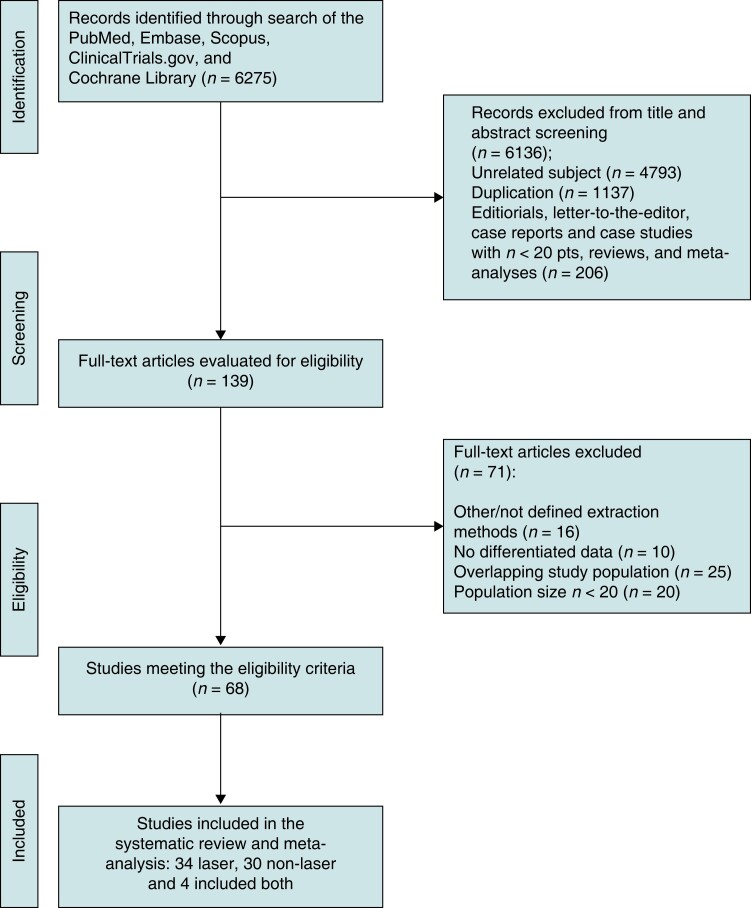
Flow chart of study identification and inclusion.

The literature search yielded 6275 results in total from PubMed, Embase Scopus ClinicalTrials.gov, and Cochrane Library. On reviewing the titles and abstracts, 6136 were excluded for unrelated subject, duplication, case studies, systematic reviews, letters, editorials, and a low study population size (*n* < 20). Full manuscripts for the remaining 139 articles were retrieved and analysed. This analysis excluded 71 articles for reasons including overlapping of data, poorly defined extraction techniques, poorly differentiated, and/or incomplete data. Subsequently, 68 studies involving 25 225 TLE procedures were included in the meta-analysis^3–6,11–74^ (*Figure 2*).

### Study characteristics

Of the 68 studies included in this meta-analysis, 65 were observational studies, 2 were randomized trials, and 1 study utilized both designs. The largest consisted of 3510 and the smallest of 24 patients. Geographically, the majority of the articles were produced by European institutions (*n* = 36), followed by North America (*n* = 21); 43 studies were from high-volume centres. Thirty studies were non-laser based (see Supplementary material online, *Table S2*), 34 were laser (see Supplementary material online, *Table S3*), and 4 included both methodologies. With respect to quality assessment, 66 observational studies were adjudicated to have had a score of ≥4 in accordance with the scale adopted by Weller *et al*. and the previous TLE meta-analyses.

After extracting data from the 68 included studies and making the relevant adjustments, there were 21 750 patients in total (non-laser 11 576; laser 10 174), pre-dominantly male (64.6%) who were 64 ± 8.1 years in age with 39 312 targeted leads of 80 ± 26.1 months of dwell time. Observed complete procedural success per lead was achieved in 95.1% of cases with 97.3% clinical success but with an occurrence of 3.9% minor complications and 1.74% major complications including 0.34% procedural mortality rate; 30-day mortality was found to be 1.7%.

### Extraction safety

**Figure 3:**
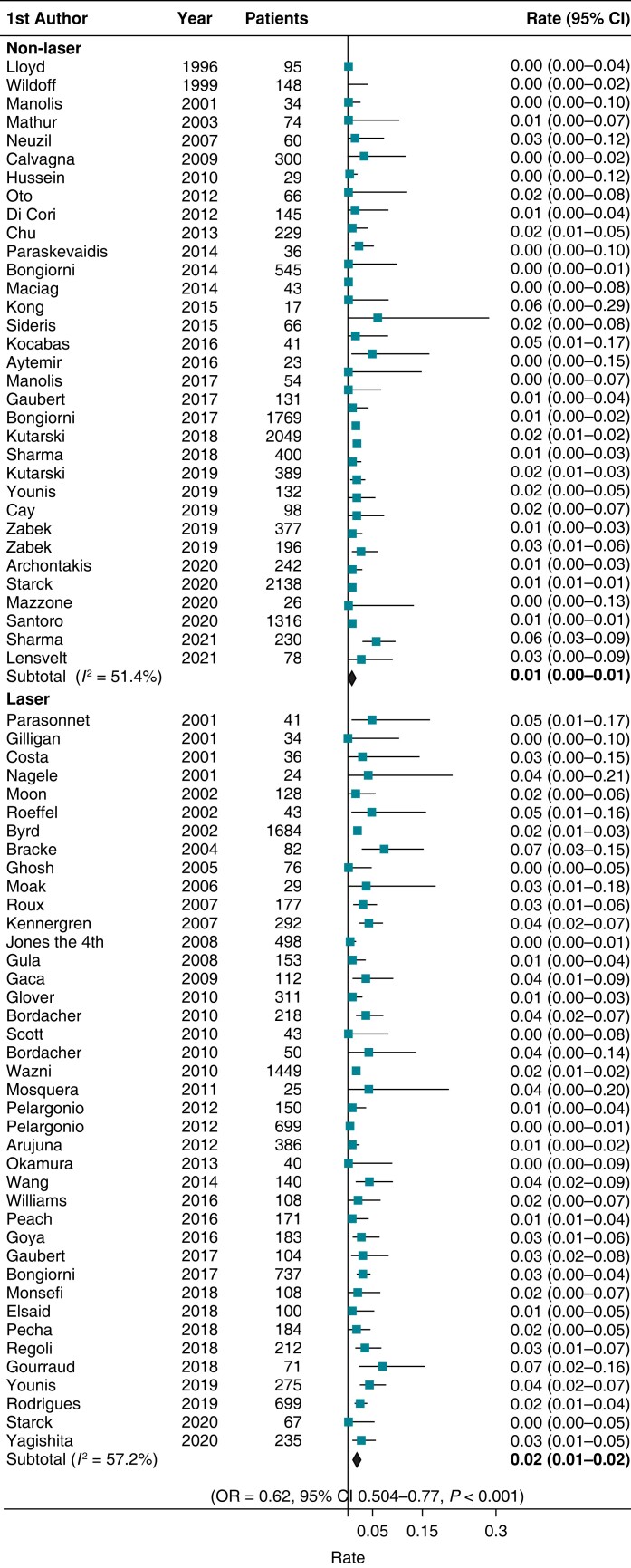
Forest plot illustrating the rate of major complications for laser and non-laser techniques. The OR corresponds to comparison of non-laser to laser techniques. CI, confidence interval; OR, odds ratio.

There was a total of 164 deaths in this study, 50 in the non-laser (see Supplementary material online, *Table S4*) and 114 in the laser groups equating to a 2.6-fold higher risk of mortality associated with laser sheath extraction [OR 2.61 (95% CI 1.9–3.6), *P* < 0.01]. Procedural-related deaths were 77, with 32 in the non-laser group and 45 in the laser cohort (see Supplementary material online, *Table S5*), which translated into a 1.6-fold greater procedural mortality with laser extraction [OR 1.6 (95% CI 1.02–2.5), *P* < 0.05]. With 147 aggregated major complications in the non-laser group and 207 with laser, non-laser technique significantly reduced the risk of major complications [OR 0.62 (95% CI 0.5–0.77), *P* < 0.01] (*Figure 3*).

After synthesizing available studies, non-laser techniques compared with laser were associated with fewer cases of both total death [pooled mean rate 0.01% (95% CI 0–0.1) vs. 0.6% (95% CI 0.3–0.9), *P* < 0.01] and procedure-related death [pooled mean rate 0% (95% CI 0–0) vs. 0.1% (95% CI 0–0.2), *P* < 0.01] (see Supplementary material online, *Figure S1*). Non-laser lead extraction comparatively to laser was associated with fewer major complications [pooled rate 0.7% (95% CI 0.4–1.1) vs. 1.7% (95% CI 1.3–2.3), *P* < 0.001] including SVC injury [pooled rate 0% (95% CI 0–0) vs. 0.5% (95% CI 0.2–0.9), *P* < 0.001] (see Supplementary material online, *Figure S2*). There was no significant difference between the groups in the prevalence of minor complications (see Supplementary material online, *Figure S3*), cardiac injury, pericardial tamponade, tricuspid injury, or bleeding events (*Table 1*).

**Table 1:** Synopsis of efficacy and safety rates of laser and non-laser techniques in lead extraction

	Non-laser aggregated rate^a^	Laser aggregated rate^a^	Non-laser pooled rate (%) (95% CI)	Laser pooled rate (%) (95% CI)	*P*-value*
Total mortality	50/11 576 (0.43%)	114/9863 (1.16%)	0.01 (0–0.1)	0.6 (0.3–0.9)	<0.01
Procedure-related death	24/11 576 (0.21%)	45/9863 (0.46%)	0 (0–0)	0.1 (0–0.2)	<0.01
Clinical success (per patient)	11 361/11 576 (98.2%)	7817/8131 (96.1%)	98.5 (97.9–99)	97.4 (96.5–98.3)	0.05
Complete success (per lead)	15 913/16 503 (96.4%)	13 470/14 405 (93.5%)	96.5 (95.7–97.3)	93.8 (92.4–95.1)	<0.01
Femoral/jugular access	1264/18 566 (6.81%)	261/8533 (3.06%)	5.6 (3.6–7.9)	2 (0.09–3.3)	<0.01
Minor complications	339/9807 (3.45%)	393/9030 (4.35%)	3.7 (2.6–5)	4.2 (2.7–5.9)	0.54
Major complications	147/11 576 (1.27%)	207/10 174 (2.03%)	0.7 (0.4–1.1)	1.7 (1.3–2.3)	<0.01
Superior vena caval injury	14/9430 (0.15%)	73/8738 (0.83%)	0 (0–0)	0.5 (0.2–0.9)	<0.01
Any cardiac injury requiring intervention	82/9430 (0.87%)	80/8738 (0.92%)	0.3 (0.2–0.5)	0.5 (0.2–0.8)	0.81
Tamponade requiring surgery	32/9807 (0.33%)	47/8738 (0.54%)	0 (0–0.3)	0.1 (0–0.3)	0.15
Tamponade with pericardiocentesis	37/9807 (0.38%)	25/8738 (0.29%)	0.01 (0–0.1)	0.03 (0–0.2)	0.94
Tricuspid valve injury with intervention	6/9807 (0.06%)	8/8738 (0.09%)	0 (0–0)	0 (0–0)	0.62
Bleeding requiring intervention	27/9807 (0.28%)	40/8738 (0.46%)	0 (0–0.1)	0.1 (0–0.3)	0.49

CI, confidence interval.

^a^Aggregated numbers for the data show different denominators according to the variable presented because of less precise classification of events in some older case series.

^*^The *P*-values are derived from pooled comparison of the non-laser and laser extraction techniques.

### Extraction efficacy

**Figure 4:**
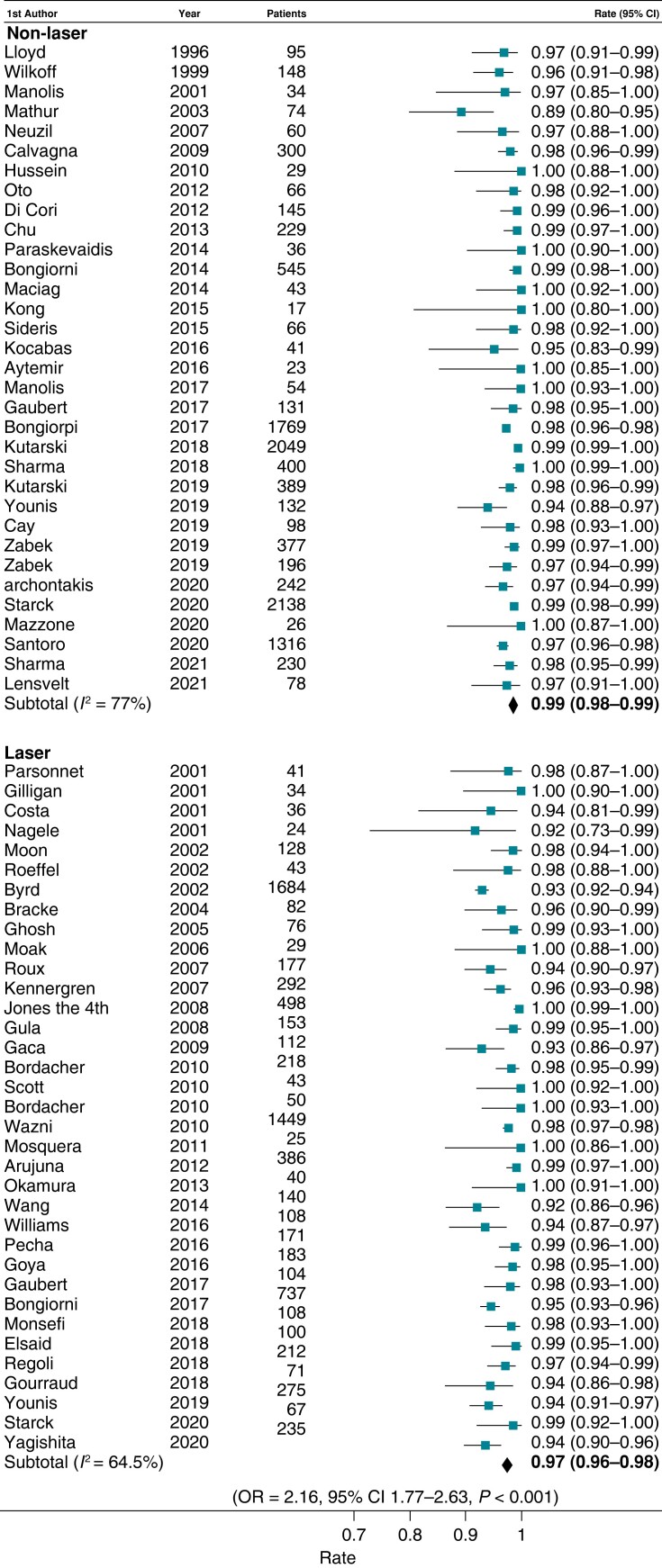
Forest plots illustrating the rate of clinical success (per patient) using non-laser and laser techniques. The OR corresponds to comparison of non-laser to laser techniques. CI, confidence interval; OR, odds ratio.

**Figure 5:**
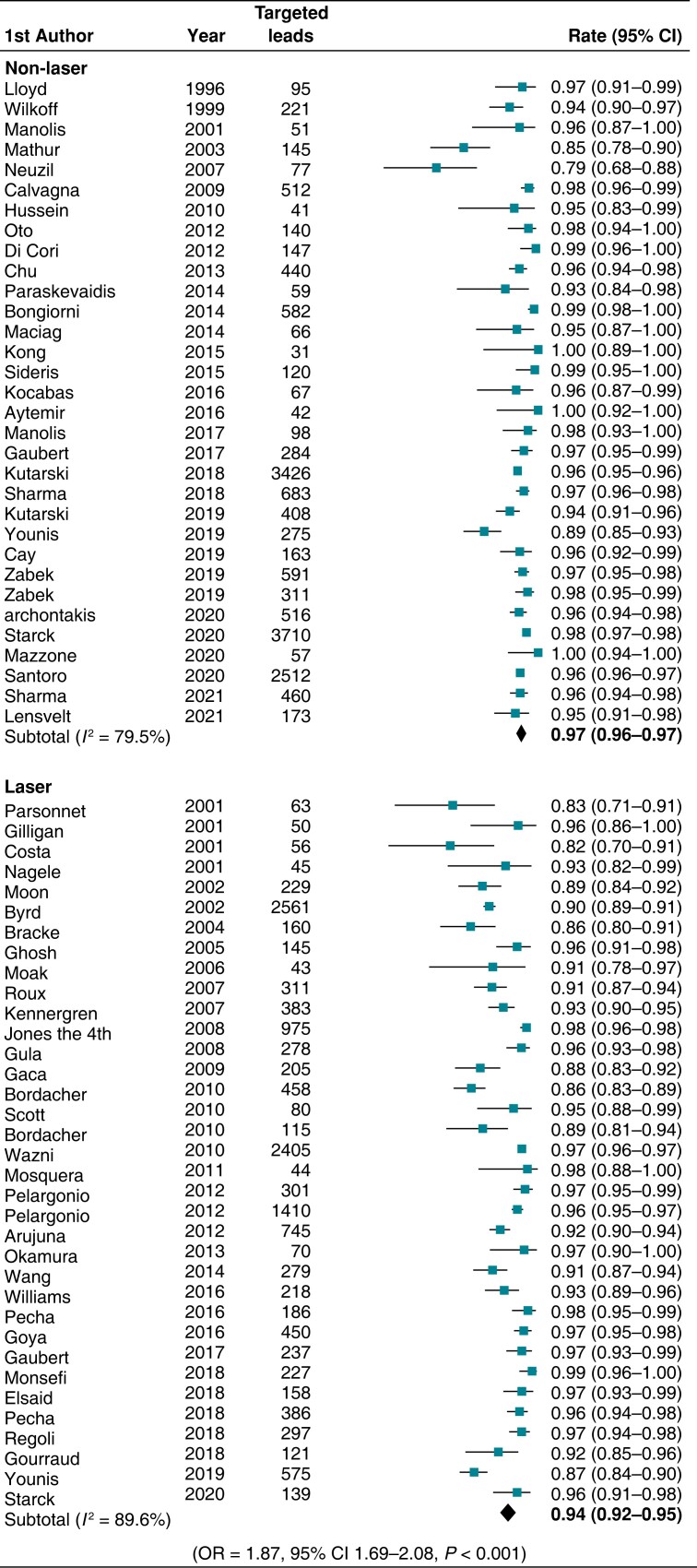
Forest plots illustrating the rate of complete success (per lead) using non-laser and laser techniques. The OR corresponds to comparison of non-laser to laser techniques. CI, confidence interval; OR, odds ratio.

Clinical success was accomplished in a total of 11 361 patients with non-laser and 7817 with laser; clinical success was more likely to be attained with non-laser compared with laser [OR 2.16 (95% CI 1.77–2.63), *P* < 0.01] (*Figure 4*). Non-laser extraction achieved complete success in 15 913 leads in comparison with laser, which achieved complete success in 13 470 leads; non-laser was significantly more likely to achieve complete success (per lead) than laser sheath [OR 1.87 (95% CI 1.69–2.08), *P* < 0.01] (*Figure 5*).

In the pooled analysis, compared with laser-based methods, non-laser extraction showed a superior rate of complete procedural success per lead [pooled rate 96.5% (95% CI 95.7–97.3, *I*^2^ = 79.5%) vs. 93.8% (95% CI 92.4–95.1, *I*^2^ = 89.6%), *P* < 0.001] and a trend towards greater clinical success per procedure [pooled rate 98.5%, (95% CI 97.9–99, *I*^2^ = 66.4%) vs. 97.4% (95% CI 96.5–98.3, *I*^2^ = 76.8%), *P* = 0.05]. The non-laser technique was associated with the use of femoral or jugular access more frequently than the laser approach [pooled rate 5.6% (95% CI 3.6–7.9, *I*^2^ = 96.5%) vs. 2% (95% CI 0.1–3.3, *I*^2^ = 93.4%), *P* < 0.01] (see Supplementary material online, *Figure S4*), although it could not be determined accurately whether this was a ‘bailout’ strategy after failure of the superior approach or a transition of choice by some operators.

### Rotational vs. Laser sheath

Restricting the analysis to compare rotational (see Supplementary material online, *Table S6*) and laser methods yielded 30/68 studies in total. From this, there were 27 procedural-related deaths of which 6 occurred in the rotational cohort and 21 in laser cases. This equated to a 2.2-fold greater risk of procedural mortality associated with laser [OR 2.24 (95% CI 0.91–5.57), *P* = 0.08]. There were 55 SVC injuries, with 6 occurring using the rotational tool and 49 with laser (*Table 2*). This signified a 5.2-fold greater risk of SVC injury associated with laser [OR 5.2 (95% CI 2.24–12.2), *P* < 0.001].

**Table 2:** Synopsis of efficacy and safety rates of rotational and laser sheath extraction in a pre-specified set of studies

	Rotational sheath aggregated rate^a^	Laser sheath aggregated rate^a^	Rotational sheath pooled rate (%) (95% CI)	Laser sheath pooled rate (%) (95% CI)	*P*-value*
Total mortality	9/3388 (0.26%)	66/5288 (1.25%)	0 (0–0.01)	0.9 (0.6–1.2)	<0.01
Procedure-related death	6/3388 (0.18%)	21/5288 (0.4%)	0 (0–0.13)	0.1 (0–0.3)	0.26
Clinical success (per patient)	3339/3388 (98.6%)	3449/3556 (97%)	99.1 (98.3–99.7)	97.4 (96.3–98.3)	0.03
Complete success (per lead)	5913/6083 (97.2%)	7657/8076 (94.8%)	97.4 (96.7–98.1)	95.0 (93.4–96.5)	<0.01
Femoral/jugular access	303/6083 (4.98%)	123/3059 (4.02%)	6 (2.6–10.4)	2.7 (0.6–6)	0.15
Minor complications	159/3388 (4.69%)	262/5288 (4.95%)	6.4 (3.8–9.0)	5.4 (3.7–7.1)	0.54
Major complications	47/3388 (1.39%)	109/5288 (2.06%)	1.2 (0.3–2.4)	1.9 (1.3–2.7)	0.66
Superior vena caval injury	6/3388 (0.18%)	49/4589(1.07%)	0 (0–0)	0.7 (0.3–1.2)	<0.01
Any cardiac injury requiring intervention	26/3388 (0.77%)	36/4589 (0.78%)	0.1 (0–0.4)	0.4 (0.1–0.9)	0.94
Tamponade requiring surgery	15/3388 (0.44%)	22/4589 (0.48%)	0 (0–0.1)	0.1 (0–0.4)	0.92
Tamponade with pericardiocentesis	6/3388 (0.18%)	14/4589 (0.31%)	0 (0–0)	0.1 (0–0.4)	0.28
Tricuspid valve injury with intervention	2/3388 (0.06%)	6/4589 (0.13%)	0 (0–0)	0 (0–0.03)	0.59
Bleeding requiring intervention	8/3388 (0.24%)	35/4589 (0.76%)	0 (0–0.1)	0.2 (0–0.7)	0.11

CI, confidence intervals.

^a^Aggregated numbers are smaller than those in *Table 1* as they are derived from a narrower data set. Denominators differ according to the variable presented because of less precise classification of events in some older case series.

^*^The *P*-values are derived from the pooled comparison of the rotational and laser extraction techniques

Pooled comparison of the rotational and laser techniques demonstrated that the rotational sheath in comparison with laser was associated with significantly lower mortality [0% (95% CI 0–0.1) vs. 0.9% (95% CI 0.6–1.2), *P* < 0.01], although there was no significant difference in the procedure-related deaths [0% (95% CI 0–0) vs. 0.1% (95% CI 0–0.3), *P* = 0.26]. The rotational tool was superior to laser in terms of clinical success [99.1% (CI 98.3–99.7) vs. 97.4% (CI 96.3–98.3), *P* = 0.03] and complete success per lead [97.4% (95% CI 96.7–98.1) vs. 95% (95% CI 93.4–96.5), *P* < 0.01]. Although there was a similar proportion of complications between the two methods, the rotational technique did have a significantly lower number of SVC injuries than laser extraction [0% (95% CI 0–0) vs. 0.7% (95% CI 0.3–1.2), *P* < 0.01] (*Table 2*).

### Meta-regression

**Figure 6:**
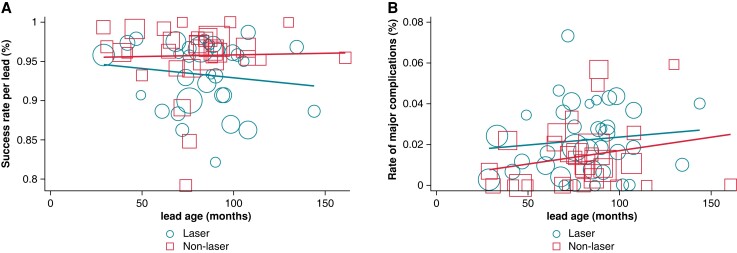
Bubble plots with random-effects meta-regression line for the association of lead age with the rate of (*A*) successful extraction and (*B*) major complications per technique (non-laser and laser) (*P* > 0.05). Bubble size is proportional to the size of each individual study.

Meta-regression analyses did not indicate a differential effect of baseline characteristics (i.e. mean age, mean prevalence of male subjects, left ventricle ejection fraction, prevalence of diabetes, CIED, and age of leads) or a potential time bias (i.e. year of study) on the rate of the main endpoints, including lead-wise extraction success (*Figure 6A*) and major complications (*Figure 6B*) conditional to the extraction technique (laser vs. non-laser) (*P* > 0.05 for all). A sensitivity analysis on newer studies (2010 onwards) showed consistent findings with the main analysis (see Supplementary material online, *Table S7*).

## Discussion

This is the largest meta-analysis comparing laser and non-laser TLE.^75,76^ Our results suggest that non-laser TLE is associated with a better level of safety and efficacy than the laser method. The findings represent a significant advancement over previous meta-analyses, whilst validating their main findings.^75,76^

Diemberger *et al*. performed a meta-analysis of TLE methods (non-laser and laser) and outcomes in the period 1998–2012, pre-dating the widespread adoption of rotational extraction tools. Their analysis therefore included relatively few procedures performed with rotational tools, representing just the early (<3 years) experience of the method.^44^ Due to the low number of mortality events in their analysis and a study design that did not compare techniques, they utilized a ‘combined’ endpoint without differentiating between mortality, minor, and major complications.

The meta-analysis of Lee *et al*. was restricted to studies comparing rotational vs. laser only, had a restricted time window (1998–2017 for laser; 2009–2017 for rotational), and did not evaluate all the significant endpoints associated with TLE, including major complications. With a 30-year time window and broad inclusion criteria, we analysed data derived from 25 225 procedures compared to the 13 674 of Diemberger *et al*. and 8869 of Lee *et al*. Those studies overlapped with each other whilst ours encompasses almost all the data included in both, excluding just the small case series (<20 cases each) that accounted for 9/62 of the papers considered by Diemberger *et al*. Case series of <20 patients were excluded from our analysis as we feared the ‘small-studies effect’; small studies are prone to publication bias, more likely to be tainted with methodological flaws and report a greater intervention effect.

Methodologically, our approach differed in that we dissected the studies that included a number of extraction techniques, parsing the data for each technique separately, providing final data sets that were as ‘clean’ as possible. We reviewed the reported complications and mortality in each study and when necessary revised the categorization of events to conform to current definitions^10^ and ensured that the classification of minor complication and major complication was consistent; adjudication of this process maintained consistency and accuracy. For instance, guidelines published in the year 2000 advised that the occurrence of a major complication should define the procedure as having been a failure^77^; in the older literature, some cases in which all components of a pacing system were extracted at the expense of a major complication were generally considered to have been successful. We corrected this inconsistency before amalgamating the data. This approach is advantageous since it attempts to reduce the risk of reporting and publication bias; it appears to have been successful in doing so as demonstrated by the symmetry in the funnel plots, suggesting improved comparability of data. We were therefore able to perform a pooled comparison of the complications, which was not possible in the previous meta-analyses, including a robust separation of events into major and minor complications. This separation was important as an index of safety and for its implication for attribution of success.

In our study, laser extraction was associated with a significantly higher rate of procedural mortality compared with non-laser methodologies, which is in keeping with the previous meta-analyses.^76^ From the aggregated deaths, we found that this is a 1.6-fold increased risk, not reported previously. Lee *et al*. did find a 9.3-fold increased risk of procedure-related mortality associated with laser when compared with the rotational sheath.^75^ Our analysis of laser vs. rotational found a 2.2-fold increased risk of death associated with laser, although failing to reach statistical significance (*P* = 0.08). The lower risk calculated in our meta-analysis could be associated with our methodology. On reviewing the included studies, we were careful to only accept deaths that occurred in the hospital and associated directly with the procedure, as ‘procedure-related’ mortality. Lee *et al*. employed a broader range; the majority of deaths occurring in their laser group were accounted for by infection (40.9%). It is difficult to ascertain the mechanism by which these deaths were influenced by extraction methodology.

Laser extraction had a significantly higher rate of SVC injury than the rotational tool, corroborating the observation of Lee *et al*. We were also able to quantify this to be 5-fold increased risk. Due to the rarity of this life-threatening complication, a significant sample size is critical in collecting enough events to provide statistical value. To reduce reporting bias, we sieved individual recruited studies for the occurrence of a severe haemothorax, which we accepted as a surrogate of SVC injury; a haemothorax causing death or requiring surgery during TLE almost always reflects an extrapericardial injury to the SVC. Our meta-analysis did not include the German Laser Lead Extraction Registry (GALLERY), as it was published after our study time frame. GALLERY was the largest laser TLE registry yet published and found a SVC injury rate of 0.83% (21/2524),^78^ whereas Patient Related Outcomes of Mechanical lead extraction Techniques (PROMET), the largest non-laser TLE study, revealed a 0% SVC injury rate.^3^ These two individual studies therefore mirror the findings of the entire meta-analysis. GALLERY demonstrated an exceptionally good rescue rate following a vascular injury (69.56%) via emergency sternotomy; perhaps because of this efficiency of surgical rescue, PROMET and GALLERY showed similar procedure-related mortality rates (0.4% and 0.55%, respectively) despite the higher rate of SVC injury in GALLERY.

The cause of SVC injury in laser-assisted extraction cannot be determined with certainty. No histological analysis is available to determine whether the disruption in the vessel is produced by burning, tearing, or cutting of the tissue. Laser energy vaporizes tissue to mobilize the leads. Laser appears to be as safe as non-laser tools in areas where the course of the lead is straight, perhaps because the sheath remains co-axial to the vessel lumen. At the subclavian SVC junction, the tip is directed towards the SVC wall as the sheath flexes reaching into the vena cava. We speculate that at this point of curvature, laser energy is directed at an angle away from the lead, potentially projecting a short distance laterally. This could extend the resulting trauma to the vessel wall, whereas a rotational or other mechanical sheath is limited in its reach as the dissecting part of the sheath cannot reach beyond the tip.

Two important advances have potentially improved TLE safety in recent years and are not well represented in this meta-analysis. The SVC occlusion balloon was introduced in mid-2016 and has been shown to improve outcome in a vena cava tear.^79^ Most of the studies included in our meta-analysis precede the widespread adoption of this technology. This may permit a reduction in the rate of mortality associated with SVC injury even below that seen in GALLERY, though SVC injury with surgical repair remains a very serious adverse event. Recent techniques designed to direct the powered sheath away from the vena cava wall have been proposed to reduce the risk of SVC injury, as well as potentially avoiding cardiac injury and improving the rate of success in TLE by either laser or non-laser methods.^80–83^

Our finding of greater effectiveness for non-laser methods (clinical and complete success) differs from that of Diemberger *et al*., who concluded that the non-laser and laser methods were similarly effective.^76^ This difference can be partly explained by temporal bias; Diemberger *et al*. only included the very early period of the first-generation rotational tool in their analysis whereas our study included the first and second generation of rotational sheaths. The rotational sheath is superior in efficacy to the laser sheath as demonstrated by our subgroup analysis and supported by the findings of Lee *et al*.^75,76^ Our study is more reflective of the current TLE landscape with the widespread use of powered sheaths, particularly rotational sheaths, but some continued use of non-powered sheath extractions. The transition to powered sheaths is neither complete nor inevitable: ELECTRa, the largest TLE study, found that simple mechanical dissecting sheaths were still commonly used (36.3%)^6^ demonstrating the continued reliance on the ‘traditional’ non-powered techniques, especially for passive leads of a long dwell time.^84^ Maintaining expertise in these methods is desirable in view of economic restrictions on healthcare in many territories.

Additional venous access (femoral or jugular) is often used as a ‘bailout’ strategy to complete extraction after the initial approach fails. It can be perceived as a surrogate marker for the incomplete success of the initial approach but may also represent a prior choice by the operator. In this analysis, the use of femoral/jugular access was more frequent in the non-laser group. This could be attributed in part to the use of femoral access as a primary extraction technique^85^ or to the planned use of multiple access sites as an deliberate strategy,^86^ particularly during the early part of the study period; TLE was in its infancy during this early period, relying on non-laser techniques from various access points. Comparing just the rotational extraction tool group to the laser group, there was no significant difference in the use of femoral or jugular access. We believe that this represents the abandonment of the multiple venous access approach with operators migrating to an approach of using the access vein only in the same way that laser operators generally do. It could also be interpreted as a reduction in the ‘bailout’ requirement following the transition from simple dilating sheaths to rotational sheaths.

The meta-regression analysis of Diemberger *et al*. found that laser sheath use was associated with a higher incidence of major complications and mortality.^76^ According to their meta-regression model, laser sheath use was evaluated as an independent variable affecting the association of extraction technique with outcomes in the total TLE population. Our meta-analysis is a pairwise comparison of the two techniques that grouped and synthesized relevant studies. Successively, our meta-regression analysis demonstrated that the principal differences in outcome between laser and non-laser methods were not attributable to baseline variables including patient age, patient gender, or lead dwell time. This is also supported by the meta-regression analysis performed by Lee *et al*., another comparative meta-analysis, which found that the difference in mortality between the laser and non-laser techniques was not affected by variates including lead age. There is compelling evidence highlighting the association of variables including lead dwell time, female gender, and patient age, with complications and procedure failure.^87–90^ These variables should affect all lead extraction procedures, irrespective of technique.

### Limitations

Limitations are inevitable in a meta-analysis of a heterogeneous literature. Most of the included studies were observational, a design that is exposed to bias but reflects real-world conditions better than a randomized trial. Some studies included in the analysis may have provided additional focus on particular complications associated with TLE, which has the potential to exacerbate bias; however, these studies fulfilled the inclusion criteria, with a consistent observational study design so were included. Effort was also made to reduce bias by homogenizing the definitions of the outcomes. The quality of the studies included in this meta-analysis was variable due to differences in study design, patient population, and methods of data collection. Efforts were made to compensate for these differences including the exclusion of studies with potential overlap, updating the outcomes to conform to current definitions and the application of meta-regression analysis. The study period was broad, so technologies were introduced and evolved over the timeframe; newer technologies may have been disadvantaged as they are represented at an earlier point in the learning curve than older methods.

## Conclusions

Both non-laser and laser TLE techniques are safe and effective. Non-laser extraction is associated with a higher success rate and a lower risk of SVC injury than laser.

## Supplementary Material

euad316_Supplementary_Data

## Data Availability

Data are on file and available upon reasonable request.
