# Supplementary material for: Comparison of non-laser and laser transvenous lead extraction: a systematic review and meta-analysis
Source: Europace. 2023 Oct 26;25(11):euad316. doi: 10.1093/europace/euad316 (PMC10638006; doi:10.1093/europace/euad316)
Supplement: euad316_Supplementary_Data [file euad316_supplementary_data.docx]

**Table 1:** Quality assessment of included studies

|  | Sharma 2021 | Lensvelt 2021 | Archontakis 2020 | Mazzone 2020 | Starck 2020 | Santoro 2020 | Yagishita 2020 | Kutarski 2019 | Zabek 2019 | Cay 2019 | Zabek 2019 |
| --- | --- | --- | --- | --- | --- | --- | --- | --- | --- | --- | --- |
| Case series from more than one centre? | 1 | 0 | 0 | 1 | 1 | 0 | 0 | 0 | 0 | 0 | 0 |
| Aim of study clearly described? | 1 | 1 | 1 | 1 | 1 | 1 | 1 | 1 | 1 | 1 | 1 |
| Case definition clearly reported? | 1 | 1 | 1 | 1 | 1 | 1 | 1 | 1 | 1 | 1 | 1 |
| Clear definition of outcomes reported? | 1 | 1 | 1 | 1 | 1 | 1 | 1 | 0 | 1 | 1 | 1 |
| Data collected prospectively? | 1 | 0 | 1 | 1 | 0 | 0 | 0 | 0 | 1 | 0 | 0 |
| Patients recruited consecutively? | 1 | 1 | 1 | 1 | 1 | 1 | 1 | 1 | 1 | 1 | 1 |
| Main findings clearly described? | 1 | 1 | 1 | 1 | 1 | 1 | 1 | 1 | 1 | 1 | 1 |
| Are outcomes stratified? | 1 | 1 | 1 | 1 | 1 | 1 | 1 | 1 | 1 | 1 | 1 |
| Total | 8 | 6 | 7 | 8 | 7 | 6 | 6 | 4 | 7 | 6 | 6 |
|  | Younis 2019 | Monsefi 2018 | Elsaid 2018 | Rodrigues 2019 | Sharma 2018 | Pecha 2018 | Regoli 2018 | Gourraud 2018 | Kutarski 2018 | Manolis 2017 | Bongiorni 2017 |
| Case series from more than one centre? | 0 | 0 | 0 | 0 | 0 | 0 | 0 | 0 | 0 | 0 | 1 |
| Aim of study clearly described? | 1 | 1 | 1 | 1 | 1 | 1 | 1 | 1 | 1 | 1 | 1 |
| Case definition clearly reported? | 0 | 0 | 1 | 1 | 1 | 0 | 1 | 1 | 1 | 1 | 1 |
| Clear definition of outcomes reported? | 0 | 1 | 1 | 1 | 1 | 0 | 1 | 1 | 0 | 0 | 1 |
| Data collected prospectively? | 1 | 0 | 0 | 1 | 0 | 0 | 0 | 1 | 0 | 1 | 0 |
| Patients recruited consecutively? | 1 | 0 | 1 | 0 | 1 | 1 | 1 | 1 | 0 | 0 | 1 |
| Main findings clearly described? | 1 | 1 | 1 | 1 | 1 | 1 | 1 | 1 | 1 | 1 | 1 |
| Are outcomes stratified? | 1 | 1 | 1 | 1 | 1 | 1 | 1 | 1 | 1 | 1 | 1 |
| Total | 5 | 4 | 6 | 6 | 6 | 4 | 6 | 7 | 4 | 4 | 7 |

|  | Gaubert 2017 | Pecha 2016 | Goya 2016 | Kocabas 2016 | Williams 2016 | Kong 2015 | Aytemir 2016 | Sideris 2015 | Paraskevaidis 2014 | Bongiorni 2014 | Wang 2014 |
| --- | --- | --- | --- | --- | --- | --- | --- | --- | --- | --- | --- |
| Case series from more than one centre? | 0 | 0 | 0 | 0 | 0 | 0 | 0 | 0 | 0 | 0 | 0 |
| Aim of study clearly described? | 1 | 1 | 1 | 1 | 1 | 1 | 1 | 1 | 1 | 1 | 1 |
| Case definition clearly reported? | 1 | 0 | 1 | 1 | 1 | 0 | 1 | 1 | 1 | 1 | 0 |
| Clear definition of outcomes reported? | 0 | 1 | 1 | 1 | 1 | 1 | 1 | 0 | 0 | 1 | 0 |
| Data collected prospectively? | 1 | 0 | 0 | 0 | 0 | 0 | 0 | 0 | 0 | 0 | 0 |
| Patients recruited consecutively? | 1 | 1 | 1 | 1 | 1 | 1 | 1 | 1 | 1 | 1 | 1 |
| Main findings clearly described? | 1 | 1 | 1 | 1 | 1 | 1 | 1 | 1 | 1 | 1 | 1 |
| Are outcomes stratified? | 1 | 1 | 1 | 1 | 1 | 1 | 1 | 1 | 1 | 1 | 1 |
| Total | 6 | 5 | 6 | 6 | 6 | 5 | 6 | 6 | 5 | 6 | 4 |

|  | Chu 2013 | Maciag 2014 | Okamura 2013 | Pelargonio 2012 | Oto 2012 | Arujuna 2012 | Di Cori 2012 | Mosquera 2011 | Glover 2010 | Bordachar 2010 | Hussein 2010 |
| --- | --- | --- | --- | --- | --- | --- | --- | --- | --- | --- | --- |
| Case series from more than one centre? | 0 | 0 | 0 | 0 | 0 | 0 | 0 | 0 | 0 | 1 | 0 |
| Aim of study clearly described? | 1 | 1 | 1 | 1 | 1 | 1 | 1 | 1 | 1 | 1 | 1 |
| Case definition clearly reported? | 1 | 0 | 0 | 1 | 0 | 0 | 1 | 0 | 0 | 1 | 1 |
| Clear definition of outcomes reported? | 0 | 0 | 1 | 1 | 1 | 1 | 0 | 0 | 0 | 1 | 1 |
| Data collected prospectively? | 0 | 0 | 0 | 0 | 0 | 0 | 0 | 0 | 0 | 1 | 0 |
| Patients recruited consecutively? | 1 | 1 | 1 | 1 | 1 | 1 | 1 | 1 | 1 | 1 | 1 |
| Main findings clearly described? | 1 | 1 | 1 | 1 | 1 | 1 | 1 | 1 | 1 | 1 | 1 |
| Are outcomes stratified? | 1 | 1 | 1 | 1 | 1 | 1 | 1 | 1 | 1 | 1 | 1 |
| Total | 5 | 4 | 5 | 6 | 5 | 5 | 5 | 4 | 4 | 8 | 6 |

|  | Wazni 2010 | Scott 2010 | Calvagna 2009 | Gaca 2009 | Gula 2008 | Jones the 4^th^ 2008 | Kennergren 2007 | Roux 2007 | Moak 2006 | Ghosh 2005 | Bracke 2004 |
| --- | --- | --- | --- | --- | --- | --- | --- | --- | --- | --- | --- |
| Case series from more than one centre? | 1 | 0 | 0 | 0 | 0 | 0 | 1 | 0 | 0 | 0 | 0 |
| Aim of study clearly described? | 1 | 1 | 1 | 1 | 1 | 1 | 1 | 1 | 1 | 1 | 1 |
| Case definition clearly reported? | 1 | 0 | 0 | 0 | 0 | 0 | 0 | 0 | 0 | 0 | 0 |
| Clear definition of outcomes reported? | 1 | 1 | 1 | 1 | 1 | 0 | 1 | 1 | 0 | 1 | 0 |
| Data collected prospectively? | 1 | 0 | 0 | 0 | 0 | 0 | 0 | 1 | 0 | 0 | 0 |
| Patients recruited consecutively? | 1 | 1 | 1 | 1 | 1 | 1 | 1 | 1 | 1 | 1 | 1 |
| Main findings clearly described? | 1 | 1 | 1 | 1 | 1 | 1 | 1 | 1 | 1 | 1 | 1 |
| Are outcomes stratified? | 1 | 1 | 1 | 1 | 1 | 1 | 1 | 1 | 1 | 1 | 1 |
| Total | 8 | 5 | 5 | 5 | 5 | 4 | 6 | 6 | 4 | 5 | 4 |

|  | Mathur 2003 | Roeffel 2002 | Moon 2002 | Byrd 2002 | Nagele 2001 | Costa 2001 | Gilligan 2001 | Parsonnet 2001 | Manolis 2001 | Lloyd 1996 | Smith 1994 |
| --- | --- | --- | --- | --- | --- | --- | --- | --- | --- | --- | --- |
| Case series from more than one centre? | 0 | 0 | 0 | 1 | 0 | 0 | 0 | 0 | 0 | 0 | 1 |
| Aim of study clearly described? | 1 | 1 | 1 | 1 | 1 | 1 | 1 | 1 | 1 | 1 | 1 |
| Case definition clearly reported? | 0 | 0 | 0 | 1 | 1 | 0 | 0 | 1 | 0 | 0 | 0 |
| Clear definition of outcomes reported? | 0 | 1 | 1 | 1 | 0 | 0 | 0 | 0 | 0 | 0 | 0 |
| Data collected prospectively? | 0 | 0 | 0 | 1 | 0 | 0 | 1 | 0 | 0 | 0 | 0 |
| Patients recruited consecutively? | 1 | 1 | 1 | 1 | 1 | 1 | 1 | 1 | 1 | 1 | 1 |
| Main findings clearly described? | 1 | 1 | 1 | 1 | 1 | 1 | 1 | 1 | 1 | 1 | 1 |
| Are outcomes stratified? | 1 | 1 | 1 | 1 | 1 | 1 | 1 | 1 | 1 | 1 | 1 |
| Total | 4 | 5 | 5 | 8 | 5 | 4 | 5 | 5 | 4 | 4 | 5 |

The Cochrane Collaboration Risk of Bias Tool applied for quality assessment of randomised trials

| Study | Adequate random sequence generation | Allocation concealment | Selective outcome reporting | Blinding of participants and personnel | Blinding of outcome assessment | Incomplete outcome data | Other biases |
| --- | --- | --- | --- | --- | --- | --- | --- |
| Bordachar 2010 | Unclear | No | Unclear | No | No | No | Unclear |
| Neuzil 2007 | Unclear | No | Yes | No | No | Yes | Unclear |
| Wilkoff 1999 | Unclear | No | Low | Unclear | No | No | Unclear |

**Table 2:** Non-Laser studies

| **1st Author** | **Year** | **Patients (n=)** | **Targeted leads (n=)** | **Age (years)** | **Male (n=)** | **LVEF (%)** | **Diabetes (n=)** | **BMI (kg/m2)** | **CIED infection (n=)** | **Lead age (months)** | **Method: traction/ locking stylet (per lead, n=)** | **Method: non-laser (per lead, n=)** | **Method: Other (femoral/ jugular, per lead, (n=)** |
| --- | --- | --- | --- | --- | --- | --- | --- | --- | --- | --- | --- | --- | --- |
| Sharma | 2021 | 230 | 460 | 64.3 | 155 | NA | 68 | 29.6 | 89 | 88.8 | 50 | 368 | 42 |
| Lensvelt | 2021 | 78 | 173 | 68 | 64 | NA | NA | 27 | 59 | 108 | 24 | 71 | 78 |
| Archontakis | 2020 | 242 | 516 | 71 | 166 | NA | NA | NA | 201 | 91.2 | 339 | 173 | 3 |
| Mazzone | 2020 | 26 | 57 | 67 | 19 | NA | NA | NA | 15 | 98.2 | 17 | 39 | 1 |
| Starck | 2020 | 2138 | 3710 | 66 | 1478 | 38.3 | NA | NA | 956 | 89.6 | 1245 | 2328 | 137 |
| Santoro | 2020 | 1316 | 2512 | 68 | 1004 | 46 | 281 | 25.4 | 864 | NA | 426 | 1872 | 214 |
| Kutarski | 2019 | 389 | 408 | 70 | 251 | NA | 98 | NA | 190 | 77 | 84 | 324 | 0 |
| Zabek | 2019 | 196 | 311 | 62.7 | 152 | NA | 66 | 27.9 | 49 | 66 | 61 | 241 | 9 |
| Cay | 2019 | 98 | 163 | 65 | 71 | 37 | 8 | NA | 55 | 64.6 | 0 | 155 | 8 |
| Zabek | 2019 | 377 | 591 | 69.2 | 215 | 49.8 | 123 | 27.8 | 69 | 106.8 | 139 | 439 | 13 |
| Younis | 2019 | 132 | 275 | 63 | 103 |  | 44 | NA | 121 | 72 | 93 | 142 | 40 |
| Sharma | 2018 | 400 | 683 | 71.2 | 286 | 37.9 | 144 | NA | 116 | 81.2 | 0 | 666 | 17 |
| Kutarski | 2018 | 2049 | 3426 | 65 | 1243 | 41.6 | 383 | NA | 815 | 86.3 | 360 | 2947 | 119 |
| Manolis | 2017 | 54 | 98 | 68.9 | 38 | NA | NA | NA | 46 | 80.4 | 60 | 27 | 1 |
| Bongiorni | 2017 | 1769 | 2859 | NA | NA | NA | NA | NA | NA | NA | 0 | NA | NA |
| Gaubert | 2017 | 131 | 284 | 68.1 | 96 | NA | NA | NA | 104 | 80.4 | 32 | 122 | 109 |
| Kocabas | 2016 | 41 | 67 | 61.5 | 30 | NA | 7 | NA | 29 | 88 | 0 | 61 | 6 |
| Aytemir | 2016 | 23 | 42 | 59.1 | 19 | 44.9 | 6 | 25.4 | 12 | 72 | 0 | 41 | 1 |
| Kong | 2015 | 17 | 31 | 67 | 10 | 65.1 | NA | NA | 17 | 130 | 0 | 30 | 1 |
| Sideris | 2015 | 66 | 120 | 64 | 46 | NA | NA | NA | 50 | 62 | 0 | 120 | 0 |
| Paraskevaidis | 2014 | 36 | 59 | 60 | 27 | NA | NA | NA | 25 | 50 | 33 | 24 | 2 |
| Bongiorni | 2014 | 545 | 582 | 62 | 465 | NA | NA | NA | 408 | 46 | 35 | 485 | 62 |
| Maciag | 2014 | 43 | 66 | 63 | 29 | NA | NA | NA | 28 | 161 | 4 | 61 | 1 |
| Chu | 2013 | 229 | 440 | 66 | 165 | NA | NA | NA | 172 | 39 | 238 | 146 | 56 |
| Oto | 2012 | 66 | 140 | 55.6 | 44 | NA | 29 | 27.4 | 39 | 85 | 0 | 133 | 7 |
| Di Cori | 2012 | 145 | 147 | 69 | 121 | 30 | NA | NA | 118 | 29 | 103 | 40 | 4 |
| Hussein | 2010 | 29 | 41 | 64.4 | 23 | NA | 9 | NA | 20 | 115 | 0 | 37 | 2 |
| Calvagna | 2009 | 300 | 512 | 67 | 218 | NA | NA | NA | 223 | 86 | 116 | 375 | 21 |
| Neuzil | 2007 | 60 | 77 | 64.6 | 46 | 63.8 | NA | NA | 59 | 74 | 0 | 77 | 0 |
| Mathur | 2003 | 74 | 145 | 58.5 | 49 | NA | NA | NA | 48 | 76 | 37 | 108 | 0 |
| Manolis | 2001 | 34 | 51 | 64 | 23 | NA | NA | NA | 21 | 42 | 10 | 31 | 10 |
| Wilkoff | 1999 | 148 | 221 | 66 | 92 | NA | NA | NA | 68 | 69 | 0 | 146 | 2 |
| Lloyd | 1996 | 95 | 95 | 65.7 | 57 | NA | NA | NA | 0 | 31 | 53 | 7 | 35 |
| Smith | 1994 | NA | 2063 | 64 | 857 | NA | NA | NA | 702 | 56 | NA | NA | 263 |

**Table 3:** Laser studies

| **1st Author** | **Year** | **Patients (n=)** | **Targeted leads (n=)** | **Age (years)** | **Male (n=)** | **LVEF (%)** | **Diabetes (n=)** | **BMI (kg/m2)** | **CIED infection (n=)** | **Lead age (months)** | **Method: traction/ locking stylet (per lead, n=)** | **Method: Laser (per lead, n=)** | **Method: Other (femoral/ jugular, per lead, (n=)** |
| --- | --- | --- | --- | --- | --- | --- | --- | --- | --- | --- | --- | --- | --- |
| Starck | 2020 | 67 | 139 | 67.7 | 50 | NA | NA | NA | 48 | 75.7 | 0 | 135 | 4 |
| Yagishita | 2020 | 235 | NA | 67 | 167 | 54 | 53 | 22.4 | 166 | 93 | NA | NA | NA |
| Younis | 2019 | 275 | 575 | 65 | 220 | NA | 117 | NA | 218 | 98.4 | 296 | 248 | 31 |
| Rodrigues | 2019 | 699 | 1630 | 70.2 | 523 | 36 | 319 | 27.5 | 111 | 33.5 | NA | 699 | NA |
| Monsefi | 2018 | 108 | 227 | 68 | 80 | 36 | NA | NA | 79 | 108 | 0 | 227 | 0 |
| Elsaid | 2018 | 100 | 158 | 60.2 | 65 | 39.6 | 29 | 29.4 | 46 | 134.4 | 0 | 146 | 12 |
| Pecha | 2018 | 184 | 386 | 66.1 | 134 | NA | 58 | NA | 126 | 99.5 | NA | 184 | NA |
| Regoli | 2018 | 212 | 297 | 69.3 | 169 | 43.4 | NA | 27.2 | 30 | NA | 53 | NA | NA |
| Gourraud | 2018 | 71 | 121 | 40 | 43 | 55 | NA | 24 | 34 | NA | 55 | 56 | 10 |
| Bongiorni | 2017 | 737 | 1250 | NA | NA | NA | NA | NA | NA | NA | 0 | NA | NA |
| Gaubert | 2017 | 104 | 237 | 69.2 | 69 | NA | NA | NA | 84 | 75.6 | 37 | 150 | 50 |
| Pecha | 2016 | 171 | 186 | 58.2 | 121 | NA | NA | NA | 92 | 46.8 | 16 | 171 | 0 |
| Goya | 2016 | 183 | 450 | 72.2 | 131 | NA | NA | 22.1 | 183 | 88.5 | 25 | 425 | 0 |
| Williams | 2016 | 108 | 218 | 67.2 | 79 | NA | 45 | 30.5 | 91 | 90 | NA | NA | NA |
| Wang | 2014 | 140 | 279 | 62.6 | 110 | NA | NA | NA | 92 | 94.8 | 0 | 279 | 0 |
| Okamura | 2013 | 40 | 70 | 65.5 | 26 | NA | NA | 21.8 | 35 | 87 | 0 | 61 | 9 |
| Pelargonio | 2012 | 699 | 1410 | 61 | 556 | 42 | 222 | NA | 657 | 29 | NA | NA | NA |
| Pelargonio | 2012 | 150 | 301 | 84 | 96 | 49 | 60 | NA | 141 | 42 | NA | NA | NA |
| Arujuna | 2012 | 386 | 745 | 67.8 | 289 | 40 | 28 | NA | 258 | 85.4 | NA | NA | NA |
| Mosquera | 2011 | 25 | 44 | 57.4 | 22 | NA | NA | NA | 8 | 84 | 19 | 25 | 0 |
| Glover | 2010 | 311 | 552 | NA | NA | NA | NA | NA | NA | 60 | NA | NA | NA |
| Bordacher | 2010 | 50 | 115 | 69 | 38 | 57 | NA | NA | 46 | 144 | 0 | 114 | 1 |
| Bordacher | 2010 | 218 | 458 | 71 | 168 | 53 | NA | NA | 182 | 108 | 0 | 452 | 6 |
| Wazni | 2010 | 1449 | 2405 | 63.4 | 1041 | 37.7 | 403 | NA | 825 | 82.1 | NA | 1449 | NA |
| Scott | 2010 | 43 | 80 | 66 | 29 | NA | NA | NA | 32 | 105.6 | 20 | 58 | 1 |
| Gaca | 2009 | 112 | 205 | 59.8 | 81 | NA | NA | NA | 66 | 69.6 | 0 | 193 | 12 |
| Gula | 2008 | 153 | 278 | 64.5 | NA | NA | NA | 28.3 | 114 | 91.2 | 85 | 193 | 0 |
| Jones the 4th | 2008 | 498 | 975 | 63.2 | 345 | NA | NA | NA | 301 | 68.4 | 218 | 702 | 55 |
| Kennergren | 2007 | 292 | 383 | 61.6 | 204 | NA | NA | NA | 131 | 74 | 0 | 375 | 8 |
| Roux | 2007 | 177 | 311 | 62 | 131 | NA | NA | NA | 88 | 93.6 | 41 | 270 | 0 |
| Moak | 2006 | 29 | 43 | 13.9 | 16 | NA | NA | NA | 0 | 49.4 | 0 | 43 | 3 |
| Ghosh | 2005 | 76 | 145 | 63 | 60 | NA | NA | NA | 50 | 102 | 0 | 142 | 3 |
| Bracke | 2004 | 82 | 160 | 62 | 52 | NA | NA | NA | 82 | 72 | NA | NA | NA |
| Roeffel | 2002 | 43 | 50 | 62 | 28 | NA | NA | NA | 26 | 67 | 27 | 23 | 0 |
| Moon | 2002 | 128 | 229 | 64 | 86 | NA | NA | NA | 52 | 61 | 68 | 157 | 4 |
| Byrd | 2002 | 1684 | 2561 | 64 | 1078 | NA | NA | NA | NA | 76 | NA | NA | 50 |
| Nagele | 2001 | 24 | 45 | NA | NA | NA | NA | NA | NA | 87.6 | 0 | 45 | 0 |
| Costa | 2001 | 36 | 56 | 54.2 | 22 | NA | NA | NA | 19 | 90 | 18 | 34 | 2 |
| Gilligan | 2001 | 34 | 50 | 64 | 34 | NA | NA | NA | 13 | 70 | 0 | 50 | 0 |
| Parsonnet | 2001 | 41 | 63 | NA | 27 | NA | NA | NA | 26 | NA | 0 | 62 | 0 |

**Table 4:** Non-Laser extraction aggregated outcomes

| **1st Author** | **Year** | **Mortality** | **Procedural Deaths (n=)** | **Minor complications (n=)** | **Major Complications (n=)** | **Complete procedural success (per lead)** | **Clinical success (per patient)** |
| --- | --- | --- | --- | --- | --- | --- | --- |
| Sharma | 2021 | 1 | 1 | 42 | 13 | 443 | 225 |
| Lensvelt | 2021 | 1 | 1 | 1 | 2 | 165 | 76 |
| Archontakis | 2020 | 2 | 0 | 7 | 2 | 496 | 234 |
| Mazzone | 2020 | 0 | 0 | 2 | 0 | 57 | 26 |
| Starck | 2020 | 3 | 2 | 63 | 20 | 3619 | 2110 |
| Santoro | 2020 | 5 | 2 | 42 | 10 | 2421 | 1273 |
| Kutarski | 2019 | 2 | 2 | 2 | 6 | 384 | 381 |
| Zabek | 2019 | 1 | 1 | 1 | 5 | 304 | 191 |
| Cay | 2019 | 1 | 1 | 7 | 2 | 157 | 96 |
| Zabek | 2019 | 0 | 0 | 8 | 4 | 572 | 372 |
| Younis | 2019 | 0 | 0 | 17 | 2 | 245 | 124 |
| Sharma | 2018 | 0 | 0 | 26 | 4 | 663 | 399 |
| Kutarski | 2018 | 8 | 6 | 32 | 36 | **3284** | 2036 |
| Manolis | 2017 | 0 | 0 | 4 | 0 | 96 | 54 |
| Bongiorni | 2017 | 16 | 6 | NA | 25 | NA | 1722 |
| Gaubert | 2017 | 0 | 0 | 7 | 1 | 276 | 129 |
| Kocabas | 2016 | 1 | 1 | 6 | 2 | 64 | 39 |
| Aytemir | 2016 | 0 | 0 | 0 | 0 | 42 | 23 |
| Kong | 2015 | 0 | 0 | 0 | 1 | 31 | 17 |
| Sideris | 2015 | 0 | 0 | 1 | 1 | 119 | 65 |
| Paraskevaidis | 2014 | 0 | 0 | 1 | 0 | 55 | 36 |
| Bongiorni | 2014 | 0 | 0 | 23 | 0 | 577 | 540 |
| Maciag | 2014 | 0 | 0 | 3 | 0 | 63 | 43 |
| Chu | 2013 | 1 | 1 | 4 | 5 | 423 | 227 |
| Oto | 2012 | 0 | 0 | 5 | 1 | 137 | 65 |
| Di Cori | 2012 | 0 | 0 | 4 | 1 | 146 | 144 |
| Hussein | 2010 | 0 | 0 | 0 | 0 | 39 | 29 |
| Calvagna | 2009 | 0 | 0 | 7 | 1 | 502 | 294 |
| Neuzil | 2007 | 0 | 0 | 4 | 2 | 61 | 58 |
| Mathur | 2003 | 0 | 0 | 12 | 1 | 123 | 66 |
| Manolis | 2001 | 0 | 0 | 0 | 0 | 49 | 33 |
| Wilkoff | 1999 | 0 | 0 | 2 | 0 | 208 | 142 |
| Lloyd | 1996 | 0 | 0 | 6 | 0 | 92 | 92 |
| Smith | 1994 | 8 | 8 | 8 | 26 | NA | NA |

**Table 5:** Laser extraction aggregated outcomes

| **1st Author** | **Year** | **Mortality** | **Procedural Deaths (n=)** | **Minor complications (n=)** | **Major Complications (n=)** | **Complete procedural success (per lead)** | **Clinical success (per patient)** |
| --- | --- | --- | --- | --- | --- | --- | --- |
| Starck | 2020 | 0 | 0 | 3 | 0 | 133 | 66 |
| Yagishita | 2020 | 4 | 0 | 5 | 6 | NA | 220 |
| Younis | 2019 | 3 | 3 | 23 | 12 | 500 | 259 |
| Rodrigues | 2019 | 5 | 5 | 44 | 17 | NA | NA |
| Monsefi | 2018 | 3 | 1 | 0 | 2 | 224 | 106 |
| Elsaid | 2018 | 0 | 0 | 1 | 1 | **153** | 99 |
| Pecha | 2018 | 4 | 0 | 2 | 3 | **371** | NA |
| Regoli | 2018 | 3 | 1 | 18 | 7 | 287 | 206 |
| Gourraud | 2018 | 0 | 0 | 10 | 5 | 111 | 67 |
| Bongiorni | 2017 | 16 | 6 | NA | 22 | NA | 697 |
| Gaubert | 2017 | 2 | 2 | 10 | 3 | 229 | 102 |
| Pecha | 2016 | 0 | 0 | 4 | 2 | 182 | 169 |
| Goya | 2016 | 4 | 1 | 7 | 5 | 437 | 180 |
| Williams | 2016 | 1 | 0 | 55 | 2 | 203 | 101 |
| Wang | 2014 | 1 | 0 | 9 | 6 | **253** | 129 |
| Okamura | 2013 | 0 | 0 | 1 | 0 | 68 | 40 |
| Pelargonio | 2012 | 5 | 1 | 19 | 2 | **1351** | NA |
| Pelargonio | 2012 | 2 | 1 | 6 | 1 | **293** | NA |
| Arujuna | 2012 | 9 | 0 | 15 | 3 | **687** | 382 |
| Mosquera | 2011 | 0 | 0 | 0 | 1 | 43 | 25 |
| Glover | 2010 | NA | NA | 53 | 3 | NA | NA |
| Bordacher | 2010 | 0 | 0 | 1 | 2 | **102** | 50 |
| Bordacher | 2010 | 2 | 2 | 0 | 8 | **395** | 214 |
| Wazni | 2010 | 27 | 4 | 44 | 24 | 2322 | 1416 |
| Scott | 2010 | 0 | 0 | 1 | 0 | 76 | 43 |
| Gaca | 2009 | 3 | 3 | NA | 4 | 181 | 104 |
| Gula | 2008 | 2 | 1 | NA | 1 | 268 | 151 |
| Jones the 4th | 2008 | 0 | 0 | 3 | 2 | 951 | 496 |
| Kennergren | 2007 | 0 | 0 | 4 | 12 | 356 | 281 |
| Roux | 2007 | 1 | 1 | 9 | 5 | 282 | 167 |
| Moak | 2006 | 0 | 0 | 3 | 1 | 39 | 29 |
| Ghosh | 2005 | 0 | 0 | 6 | 0 | 139 | 75 |
| Bracke | 2004 | 3 | 2 | NA | 6 | 138 | 79 |
| Roeffel | 2002 | 0 | 0 | 5 | 2 | NA | 42 |
| Moon | 2002 | 0 | 0 | 3 | 2 | 203 | 126 |
| Byrd | 2002 | 13 | 10 | 24 | 31 | 2305 | **1566** |
| Nagele | 2001 | 1 | 1 | NA | 1 | 42 | 22 |
| Costa | 2001 | 0 | 0 | NA | 1 | 46 | 34 |
| Gilligan | 2001 | 0 | 0 | 4 | 0 | 48 | 34 |
| Parsonnet | 2001 | 0 | 0 | 1 | 2 | 52 | 40 |

**Table 6:** Rotational tool studies

| **1st Author** | **Year** | **Patients (n)** | **Targeted leads (n=)** | **Age (years)** | **Male (n=)** | **LVEF (%)** | **Diabetes (n=)** | **BMI (kg/m2)** | **CIED infection (n=)** | **leads age (months)** | **Method: traction/ locking stylet (per lead, n=)** | **Method: non-laser (per lead, n=)** | **Method: Other (femoral/ jugular, per lead, (n=)** |
| --- | --- | --- | --- | --- | --- | --- | --- | --- | --- | --- | --- | --- | --- |
| Sharma | 2021 | 230 | 460 | 64.3 | 155 | NA | 68 | 29.6 | 89 | 88.8 | 50 | 368 | 42 |
| Lensvelt | 2021 | 78 | 173 | 68 | 64 | NA | NA | 27 | 59 | 108 | 24 | 71 | 78 |
| Archontakis | 2020 | 242 | 516 | 71 | 166 | NA | NA | NA | 201 | 91.2 | 339 | 173 | 3 |
| Mazzone | 2020 | 26 | 57 | 67 | 19 | NA | NA | NA | 15 | 98.2 | 17 | 39 | 1 |
| Starck | 2020 | 2138 | 3710 | 66 | 1478 | 38.3 | NA | NA | 956 | 89.6 | 1245 | 2328 | 137 |
| Cay | 2019 | 98 | 163 | 65 | 71 | 37 | 8 | NA | 55 | 64.6 | 0 | 155 | 8 |
| Sharma | 2018 | 400 | 683 | 71.2 | 286 | 37.9 | 144 | NA | 116 | 81.2 | 0 | 666 | 17 |
| Kocabas | 2016 | 41 | 67 | 61.5 | 30 | NA | 7 | NA | 29 | 88 | 0 | 61 | 6 |
| Kong | 2015 | 17 | 31 | 67 | 10 | 65.1 | NA | NA | 17 | 130 | 0 | 30 | 1 |
| Aytemir | 2016 | 23 | 42 | 59.1 | 19 | 44.9 | 6 | 25.4 | 12 | 72 | 0 | 41 | 1 |
| Oto | 2012 | 66 | 140 | 55.6 | 44 | NA | 29 | 27.4 | 39 | 85 | 0 | 133 | 7 |
| Hussein | 2010 | 29 | 41 | 64.4 | 23 | NA | 9 | NA | 20 | 115 | 0 | 37 | 2 |

**Table 7**: Sensitivity analysis for efficacy and safety rates of laser and non-laser techniques in lead extraction in studies newer than 2009

|  | **Non laser**  **Pooled proportion (95% CI)** | **Laser**  **Pooled proportion (95% CI)** | **P for interaction** |
| --- | --- | --- | --- |
| Total mortality | 0.01 (0-0.091) | 0.99 (0.683-1.34) | **<0.001** |
| Procedural mortality | 0 (0-0.001) | 0.012 (0.2-0.264) | **0.023** |
| Clinical success (per patient) | 98.8 (98.2-99.3) | 97.5 (96.4-98.4) | **0.028** |
| Complete success (per lead) | 97 (96.3-97.7) | 94.9 (93.3-96.3) | **0.011** |
| Major complications | 0.844 (0.479-1.28) | 1.76 (1.19-2.41) | **0.045** |

**Figure 1:** Forest plot demonstrating the rate of procedural-mortality in Non-laser and Laser techniques.


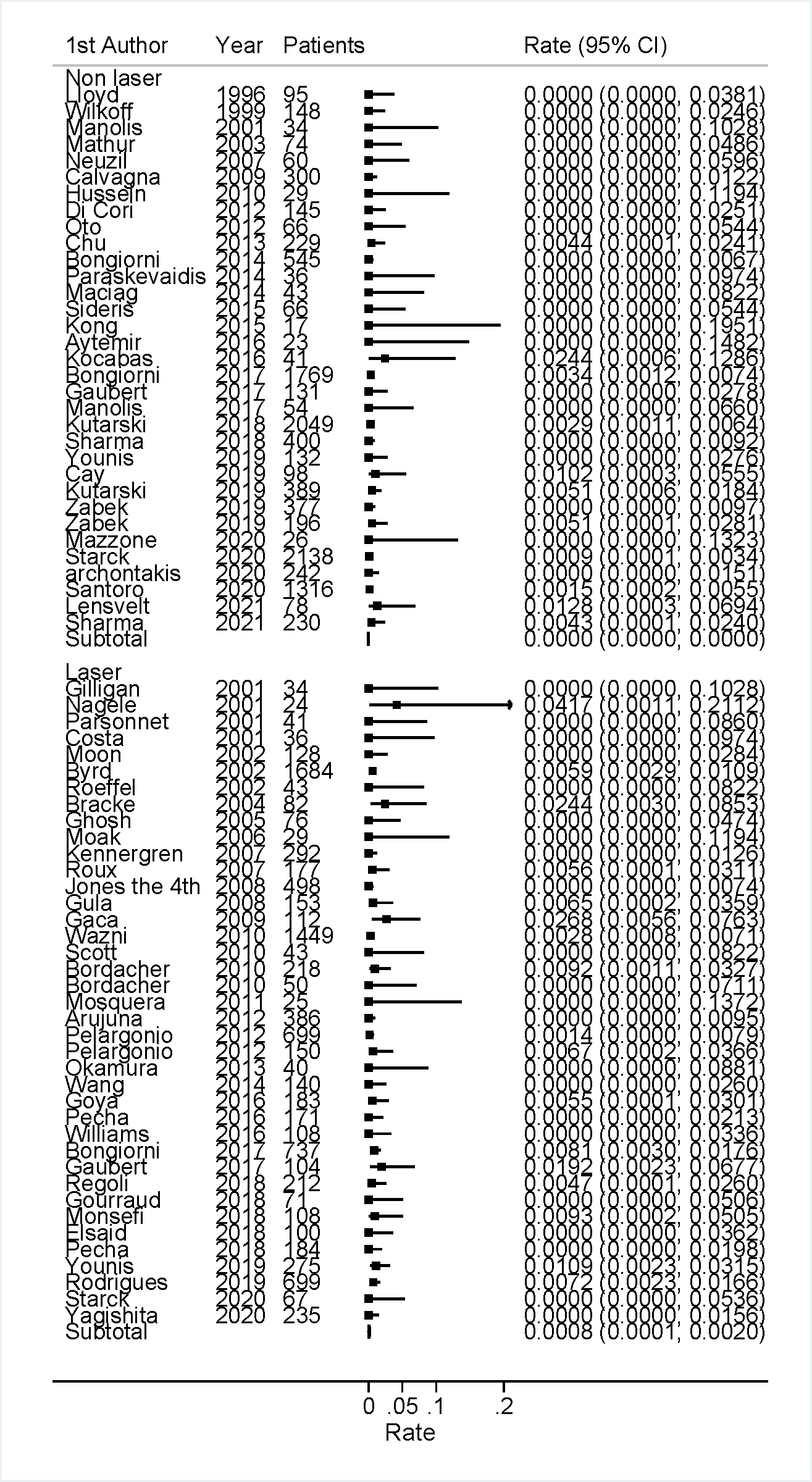


Laser assisted lead extraction was associated with higher rate of procedural mortality than non-laser (p<0.01)

**Figure 2:** Forest plot demonstrating the rate of superior vena cava injury in Non-laser and Laser techniques


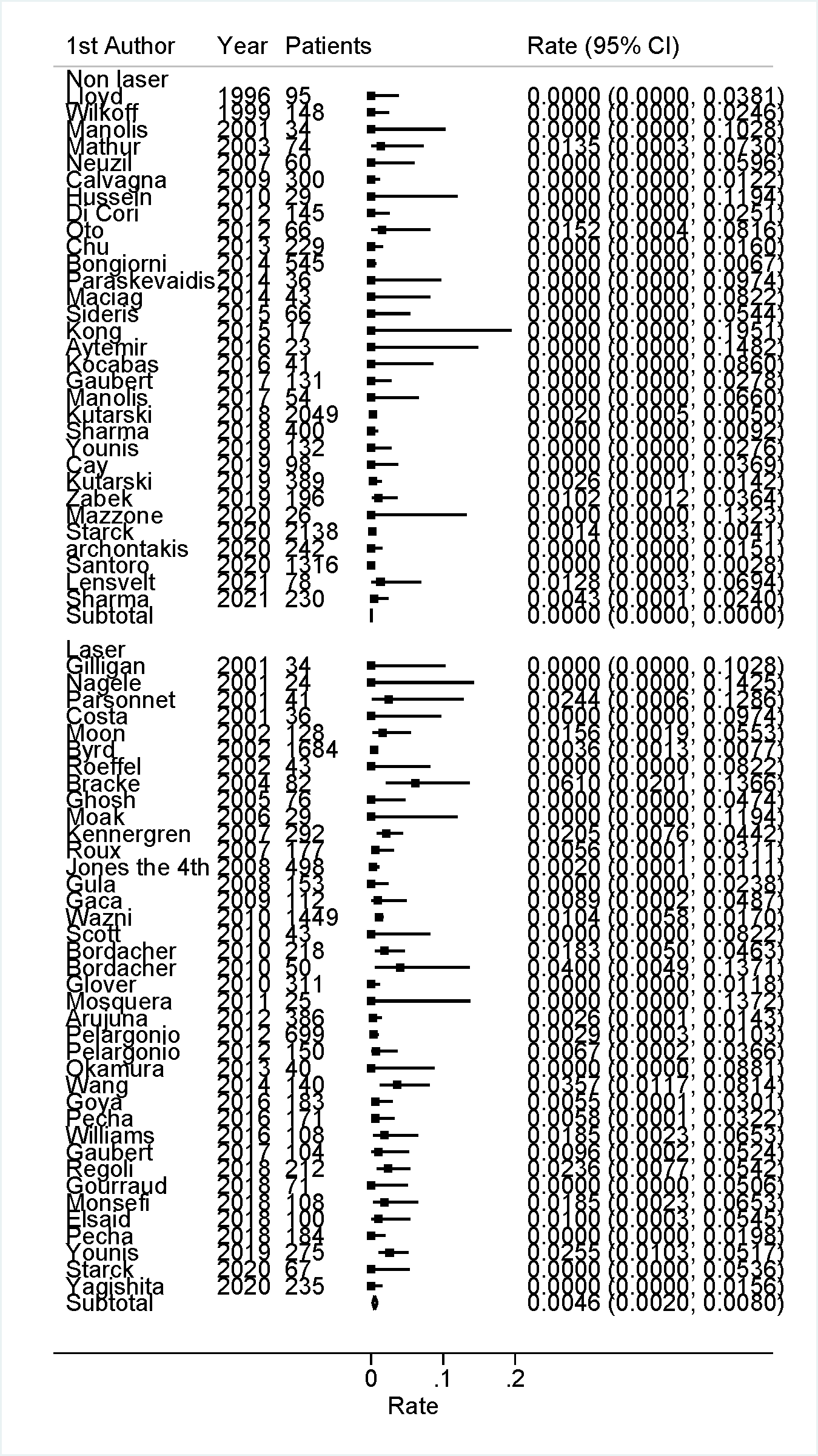


There was a higher rate of superior vena cava associated with the Laser sheath in comparison to the non-laser techniques (p<0.01)

**Figure 3:** Forest plot demonstrating the rate minor complications in Non-laser and Laser techniques.


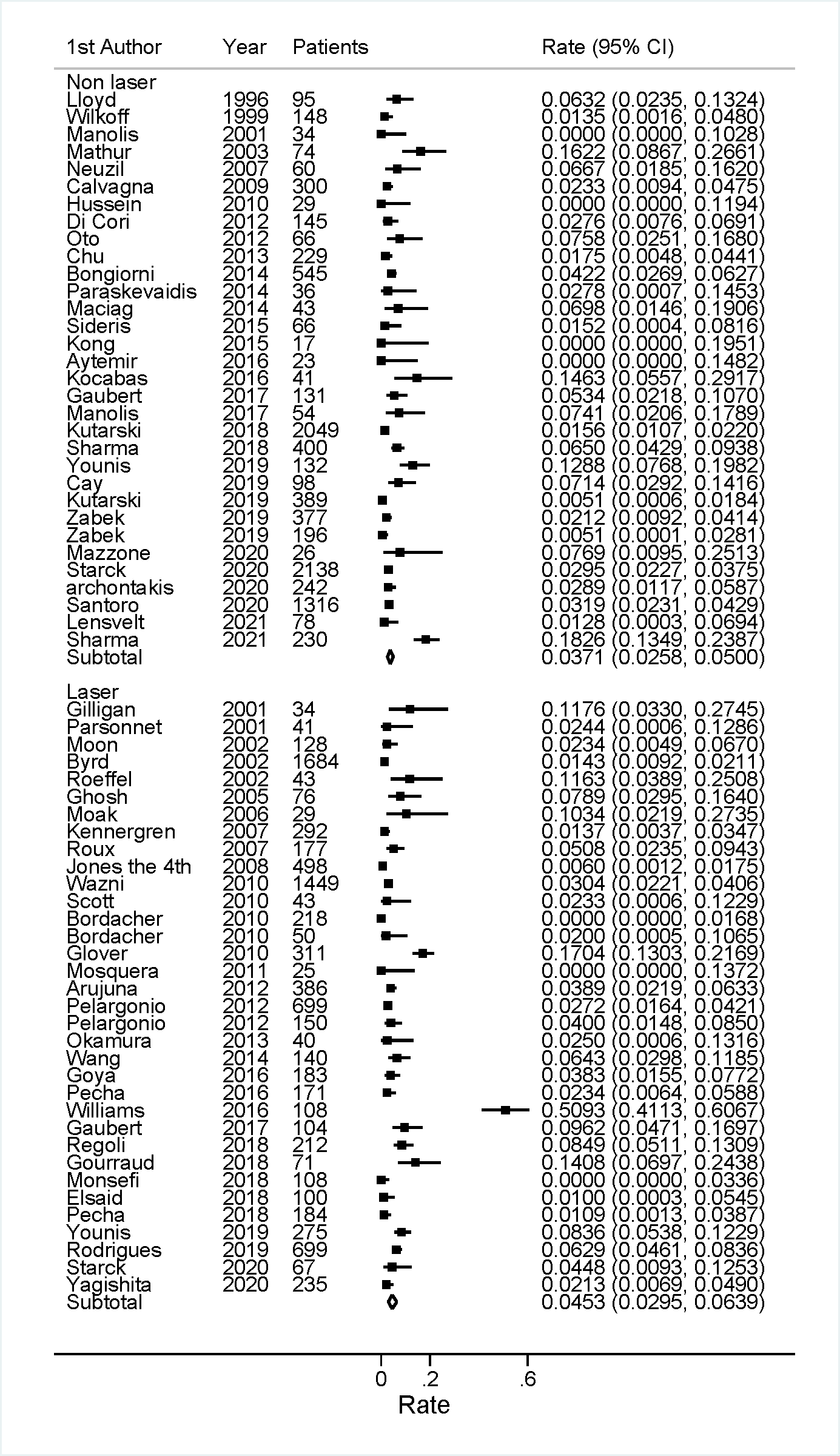


There was no significant difference in the rate of minor complications between non-laser and Laser transvenous lead extraction (p=0.54)

**Figure 4:** Forest plot summarising the rate of femoral or jugular use in non-laser and Laser lead extraction


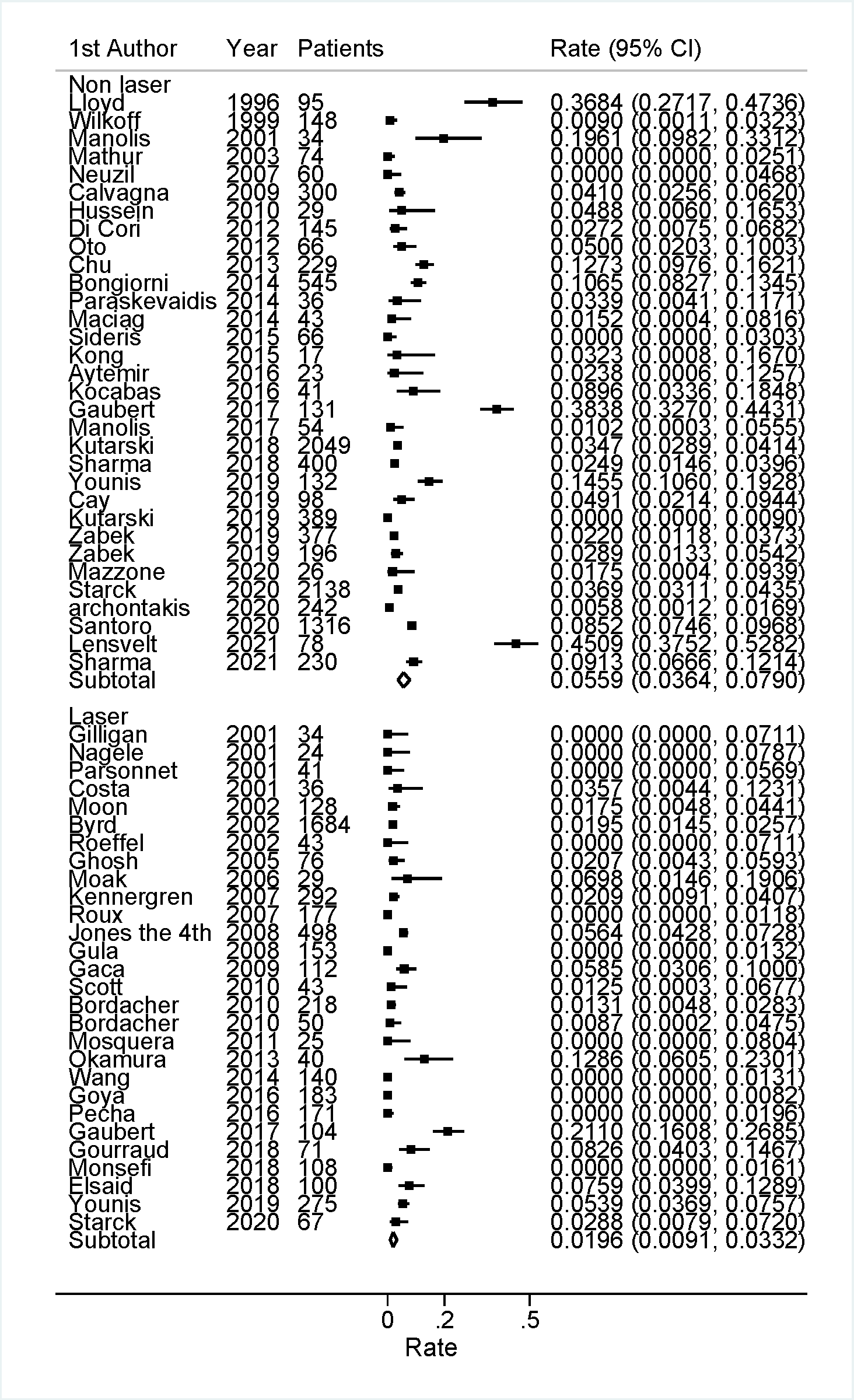


Non-laser lead extraction was associated with higher use of the femoral/jugular access for lead extraction than the laser approach (p<0.01)
